# Correlated Evolution of Positions within Mammalian cis Elements

**DOI:** 10.1371/journal.pone.0055521

**Published:** 2013-02-08

**Authors:** Rithun Mukherjee, Perry Evans, Larry N. Singh, Sridhar Hannenhalli

**Affiliations:** 1 Computational Biology Program, Fred Hutchinson Cancer Research Center, Seattle, Washington, United States of America; 2 Department of Pathology, School of Medicine, Yale University, New Haven, Connecticut, United States of America; 3 Genetic Diseases Research Branch, NHGRI, NIH, Bethesda, Maryland, United States of America; 4 Center for Bioinformatics and Computational Biology, Department of Cell and Molecular Biology, University of Maryland, College Park, Maryland, United States of America; University of Leuven, Belgium

## Abstract

Transcriptional regulation critically depends on proper interactions between transcription factors (TF) and their cognate DNA binding sites. The widely used model of TF-DNA binding – the *Positional Weight Matrix* (*PWM*) – presumes independence between positions within the binding site. However, there is evidence to show that the independence assumption may not always hold, and the extent of interposition dependence is not completely known. We hypothesize that the interposition dependence should partly be manifested as correlated evolution at the positions. We report a *Maximum-Likelihood* (*ML*) approach to infer correlated evolution at any two positions within a PWM, based on a multiple alignment of 5 mammalian genomes. Application to a genome-wide set of putative *cis* elements in human promoters reveals a prevalence of correlated evolution within *cis* elements. We found that the interdependence between two positions decreases with increasing distance between the positions. The interdependent positions tend to be evolutionarily more constrained and moreover, the dependence patterns are relatively similar across structurally related transcription factors. Although some of the detected mutational dependencies may be due to context-dependent genomic hyper-mutation, notably CG to TG, the majority is likely due to context-dependent preferences for specific nucleotide combinations within the *cis* elements. Patterns of evolution at individual nucleotide positions within mammalian TF binding sites are often significantly correlated, suggesting interposition dependence. The proposed methodology is also applicable to other classes of non-coding functional elements. A detailed investigation of mutational dependencies within specific motifs could reveal preferred nucleotide combinations that may help refine the DNA binding models.

## Introduction

Eukaryotic gene transcription is tightly regulated, in large part, by transcription factor proteins (TF) that bind to DNA, often in a sequence-specific fashion [1], [2]. The DNA-binding preference of a TF is determined using a variety of *in vitro* and *in vivo* approaches [3], and is commonly represented by a *Positional Weight Matrix (PWM)* [4]. A PWM is a 4-by-n matrix where the rows correspond to the 4 bases, and the columns correspond to *n* positions in the binding site. Each column indicates the preference for the 4 bases at a specific position. Although the PWM is currently used as the *de facto* model of TF-DNA interaction, a major shortcoming of this model is the assumption that the nucleotide preferences at individual positions within the binding site are independent of each other. However, there are both direct experimental evidence [5], [6], as well as indirect evidence based on computational modeling [7], [8], that suggest that the interposition independence assumption does not hold universally. The extent and nature of interposition dependence is not completely known, and it has been argued that overall, a simple additive (assuming independence between positions) model may be sufficient to capture the TF-DNA interaction [9]. However, our focus here is on detecting the specific instance of inter-positional dependence and not on the extent to which these dependencies affect the overall accuracy of binding site prediction.

In any biological system with interdependent components, a mutation in one component may lead to a compensatory change in other interacting components. Compensatory changes and co-evolution of functionally interacting components have been previously demonstrated in several contexts [10], [11], [12], [13], [14], [15], [16]. In the context of TF elements, several previous studies have assessed interposition dependence by computing the correlation between nucleotides at two positions [17], [18]. However, these studies are based on instances of the DNA element only within a single species. A more direct approach to assess interposition dependence is to compare the histories of nucleotide substitutions at the two positions [19]. Specifically, if a mutation at position *i*, say from nucleotide *u* to *v*, frequently coincides with a mutation at position *j*, say from nucleotide *x* to *y*, such correlated evolutionary patterns can serve as a reasonable proxy for dependence between positions *i* and *j*.

Here we present a novel *Maximum-Likelihood* (*ML)* approach to quantify co-evolution of pairs of positions within a TF binding motif. Our analysis is based on putative binding sites for 64 vertebrate TFs within human proximal promoters. We infer evolutionary patterns from genome-scale alignments of Human, Chimpanzee, Rat, Mouse, and Dog. We found that interposition dependence is highly prevalent, especially between adjacent positions within binding sites. Typically a TF residue interacts only with a few adjacent DNA bases [20], [21]. Accordingly, we found a trend of decreasing dependence with increasing distance between the two positions. The interdependent positions are evolutionarily more constrained than the positions that are independent. Moreover, we found that the interposition dependence pattern is relatively similar among the structurally related TFs, suggesting a structural basis for these patterns. We discuss a few cases of interdependent positions in the context of solved structures of DNA-bound TFs. In summary, our work presents compelling additional evidence to support co-evolution, and thus interdependence, between positions within mammalian *cis* elements.

## Results

### Overview of the Data and the Approach

**Figure 1:**
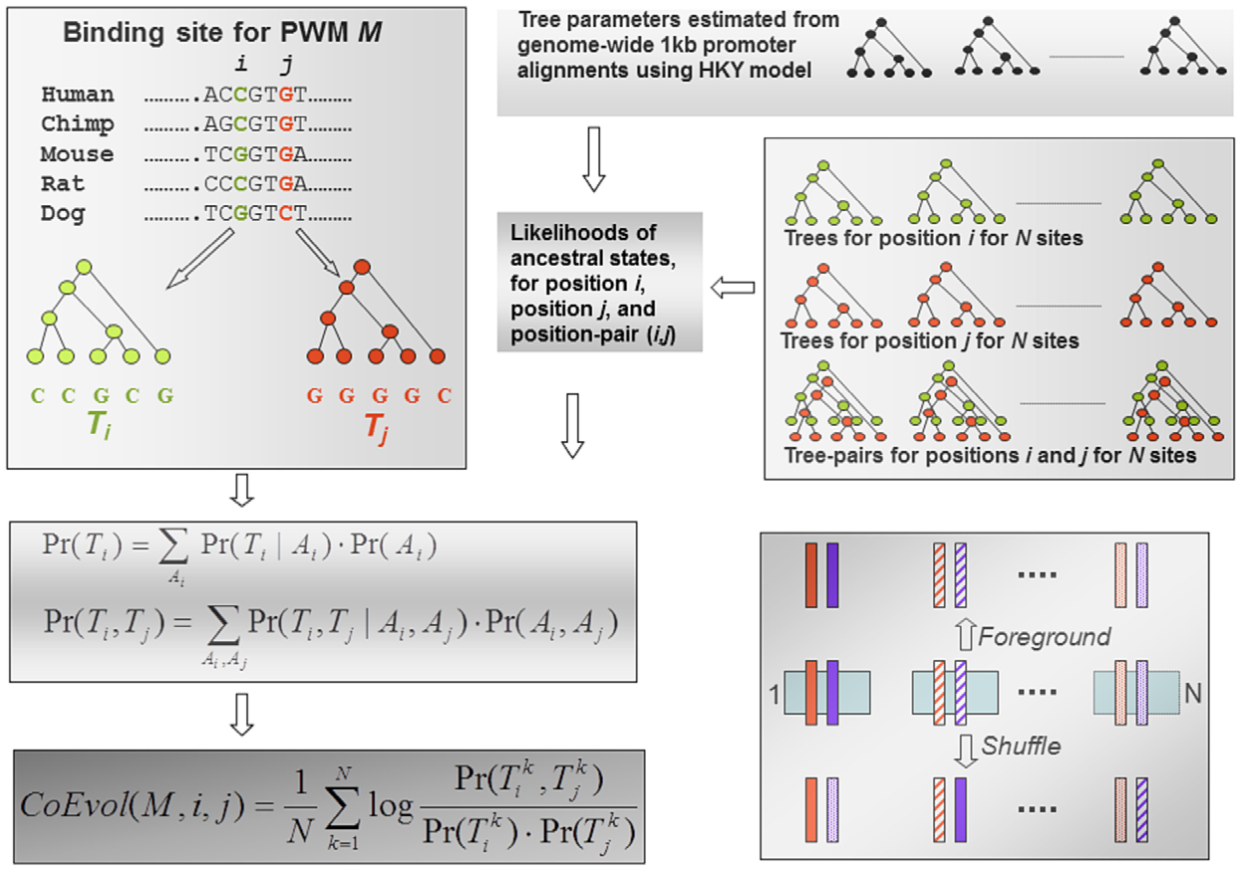
Overview of the approach. The top left panel depicts positions *i* and *j* within a binding site for a PWM *M* having *N* genome-wide matches. The binding site in human is shown in the context of a 5-species multiple alignment. The top left panel also shows the phylogenetic trees for the two positions. The phylogenetic parameters are estimated from the genome-wide set of promoter alignments (top right panel). The likelihoods of ancestral nodes for a specific PWM and position (or position-pair) are then estimated from the *N* instances and the phylogenetic parameters. The likelihoods of individual trees and tree-pairs and ultimately the *CoEvol* for a pair of PWM positions are then estimated as detailed in the text. The lower right panel illustrates the procedure to generate *Shuffle* control. The figure depicts *N* instances of position pair (*i,j*) in the central row. A random *j*-position is paired with each of the *i*-positions (lower row).

Figure 1 illustrates our approach. Our analysis is based on a genome-wide set of putative TF binding sites in human proximal promoters based on 64 vertebrate PWMs in JASPAR [22] (see Table S1 for the list of PWMs). We only consider binding sites contained within gapless regions in the multiple alignment of 5 species – Human, Chimpanzee, Mouse, Rat, and Dog, obtained from the UCSC database [23] (see Additional File in Supplementary Data for all binding sites for all PWMs used in our study). Consider PWM *M, L* bases long, and with *N* binding sites in the human promoters. For each pair of positions (*i,j*), *1≤ i<j ≤ L*, our *Foreground* set includes *N* pairs of multiple alignment columns corresponding to the positions *i* and *j* in the *N* binding sites. For each position-pair *(i,j)* in the *Foreground* set, we computed *CoEvol(i,j)* to quantify the extent to which positions *i* and *j* co-evolve and thus can be deemed interdependent. We compare the *CoEvol* estimated from the *Foreground* set with that for the following three control sets, each similarly consisting of *N* pairs of positions.

1. **Random**: For each PWM motif *M*, with length *L*, and with *N* binding sites in the genome, we randomly selected *L* positions *N* times from gapless positions of the multiple alignment corresponding to the human promoters. Each set of *L* positions was concatenated and treated as a ‘binding site’. The total of *N* random sites thus generated were then treated identically to the *Foreground* to estimate *CoEvol* for all (*i,j*) position pairs. This control represents the baseline expectation.
2. **RandomContext**: This control is meant to capture the context-dependent mutational variations in the genome. The well-known CG to TG hyper-mutability is an extreme example of this phenomenon. There may be other reasons for local dependence in the promoter that needs to be distinguished from the inter-positional dependence within TF binding sites. For this control, we randomly selected *N* sites, each *L* bases long from gapless aligned portion of the promoter, similar to the *Foreground*.
3. **Shuffle**. While using the same L*N positions as in *Foreground*, we pair each of the *N i*-position instances randomly with one of the *N j*-position instances (without replacement) (Figure 1). In other words, we construct each of the *N* random binding sites by selecting each of the *L* positions randomly from one of the *N* instances of that position. This procedure breaks the contextual link between (*i,j*) instances while still preserving the independent compositional and species-specific properties of the two position sets.

### A Sizable Fraction of Position-pairs within Transcription Factor Binding Sites have Co-evolved

**Figure 2:**
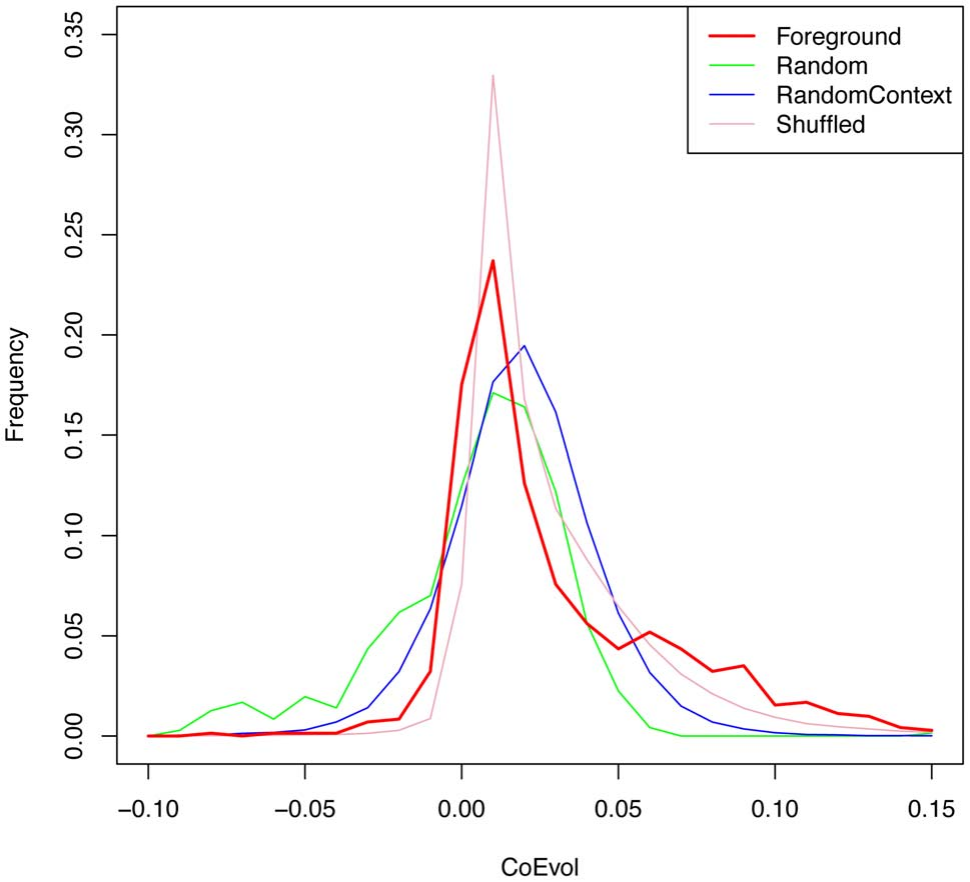
The distribution of *CoEvol* values for scope 1. Shown are *CoEvol* values for *Foreground*, *Random*, *RandomContext* and *Shuffle* controls. Values for *RandomContext* are included after correcting for a shift in the distribution of tree likelihoods (see Methods for details). Relative to the most stringent control – *Shuffle*, the *Foreground CoEvol* values are significantly greater (Mann-Whitney U test p-value = 0.02, Kolmogorov-Smirnov test p-value = 1.1e-12).

In each of 4 sets – *Foreground*, *Random*, *RandomContext*, and *Shuffle*, for each motif *M*, and for each position pair (*i,j*) we computed *CoEvol(i,j)*. The random expectation of *CoEvol* is zero, as evident for *Random* control. A positive value may indicate correlated evolution. We define *scope* as |*i-j*|. For each scope *s*, we pooled all *CoEvol(i,i+s)* for all *i* and all *M*. For scope = 1, Figure 2 shows the *CoEvol* distributions in all sets. For technical reasons, we had to devise a special procedure to deal with *RandomContext* – see Methods for details. As expected, the *CoEvol* values for *RandomContext* are significantly greater than 0, suggesting interdependence between adjacent positions in the promoter regions regardless of TF binding, as noted previously [24]. The *Shuffle* control comes closest to the *Foreground*, indicating that the underlying compositional and evolutionary patterns of the two positions greatly contribute to *CoEvol* values in the *Foreground*. Nevertheless, even relative to this most stringent *Shuffle* control, the *Foreground* has higher values of *CoEvol*. We regenerated the *Shuffle* control set 100 times and pooled all *CoEvol* values to test the null hypothesis that the *CoEvol* values for the *Foreground* are no greater than that for the *Shuffle*. We found that the *Foreground CoEvol* values for scope 1 are significantly greater than that for the pooled *Shuffle* values (Mann-Whitney U test p-value = 0.02, Kolmogorov-Smirnov test p-value = 1.1e-12). The corresponding p-values for scope 1 when comparing *Foreground* versus *RandomContext* were 6.1e-4 and 0, respectively.

We repeated the Mann-Whitney test for each scope from 1 through 8 comparing *Foreground* and the pooled *Shuffle CoEvol* values. The p-values were significant only for scopes 1, 2, and 3 and not so for greater scopes. Thus we find that the *CoEvol* signal is limited to lower scopes. It is possible that at higher scopes, only a small but significant number of position-pairs co-evolve, and will not be detected by a global test of differences in distribution. Also, the median binding site length for our set of 64 PWMs is 12; so higher scopes are only relevant to a fraction of the PWMs. For these reasons, we restrict our analysis to scopes 1–8.

To measure differences between *Foreground* and *Shuffle* in the right tail of the distribution, we adopted the following strategy. Using *Random* as the negative control, we computed *T^99^* as the 99^th^ percentile threshold of all *CoEvol* values for the *Random* control. Let *F^S^* represent the fraction of all *Foreground CoEvol* values for scope *s* that are greater than *T^99^*. The expected value of *F^S^* is 1%. However, we observed that in all scopes, 1≤ *s* ≤8, *F^S^* ranged from 17% to 25%. Similarly, we computed *S^S^*, defined as the fraction of all *Shuffle CoEvol* values for scope *s* that are greater than *T^99^*. To estimate the significance of *F^S^* relative to *S^S^*, we computed the fraction of 100 *Shuffle* sets in which *S^S^* ≥ *F^S^*. For scopes 1 through 7, none of the 100 *Shuffle* sets had *S^S^* ≥ *F^S^*, corresponding to a nominal p-value <0.01. For scope 8 the p-value was 0.04. Thus, based on the right tail analysis, there is significantly greater enrichment of high *CoEvol* values in the *Foreground* relative to the *Shuffle* control in all scopes.

### The Number of Interdependent Position Pairs Decreases with Increasing Scope

For a motif *M* with *N* binding sites, and for a specific position pair (*i,j*), let *CE^F^* be the *CoEvol* value in the *Foreground*. *CE^S^* is defined similarly for the *Shuffle*. We estimate the significance of *CE^F^* as the fraction of 100 shuffles for which *CE^S^* ≥ *CE^F^.* Given the p-values for all *CoEvol(i, j)* (all position pairs for each PWM), we estimate their q-values (False Discovery Rate) using the Storey-Tibshirani method to control for multiple testing [25]. All *CoEvol(i, j)* with a q-value ≤ 0.05 are considered to represent instances of co-evolution. Out of a total of 3914 position pairs evaluated for all motifs and for all scopes, 315 were deemed significant with an estimated false discovery rate of 5% (see Figure S1 and Table S2). Of these 315, 92 were in scope 1, monotonically decreasing to 23 for scope 8. An alternative measure of interposition dependence, and one that does not rely on co-evolution, is the mutual information between the nucleotide probability distributions at the two columns of the PWM. We found that the 315 position-pairs that our method deemed to be co-evolving have significantly greater mutual information relative to all other position-pairs (Mann-Whitney U test p-value = 8.7e-06).

### Interdependent Positions have Greater Evolutionary Conservation

The 315 interdependent position-pairs correspond to 353 unique PWM positions. There are 830 other positions for the 64 PWMs that were not deemed co-evolving with any other position. Thus, the detected position pairs are not dominated by a few positions, and include a large fraction of positions. We compared the 353 interdependent positions with the 830 independent positions with respect to their compositional and conservation properties. We did not find a significant difference in the C+G content between the interdependent and independent positions. To estimate the evolutionary conservation for the *i^th^* position of PWM *M* with *N* binding sites in the genome, we extracted the 17-species *Phastcons* evolutionary conservation score from Galaxy (main.g2.bx.psu.edu) and averaged over the *N* instances of position *i*. Our choice of 17-species alignment to estimate conservation is meant to minimize the dependence on the input set of alignment based on 5 mammalian species. We found that the interdependent positions tend to be evolutionarily more conserved (Mann-Whitney U test p-value = 3.5e-4). We note that there is no *a priori* bias in our method’s ability to detect co-evolution towards greater evolutionary conservation. Our results thus suggest that the interdependent positions are under a greater constraint against mutations.

### Structurally Related TFs Exhibit Similar Patterns of Interposition Dependence

For a pair of PWMs *M1* and *M2*, we quantified the similarity *J(M1,M2)* in their interposition dependence as follows. A position-pair (*i1, j1*) for *M1* is considered to “match” a position-pair (*i2, j2*) for *M2* if (1) *j1-i1 = j2-i2*, i.e., they have the same scope, and (2) |*i1-i2*| ≤ D, where the parameter *D* allows for a shift between the positions in the two PWMs. *J(M1,M2)* is then defined as the ratio of the number of matching interdependent position-pairs between the two PWMs and the total number of interdependent position-pairs for the two PWMs; this is analogous to the standard Jaccard index. We grouped PWMs according to the TF’s structural class annotated in TRANSFAC. We compared, using the Mann-Whitney U test, the 98 *J(M1,M2)* values corresponding to PWM pairs within the same family, with the 1498 *J(M1,M2)* values corresponding to PWM pairs in different families. We found that the within-family similarity was significantly greater than the cross-family similarity with p-values of 2.9e-6, 1.4e-6, and 1.7e-8 for D = 0, 1, and 2 respectively. This suggests that structurally related TFs tend to exhibit similar patterns of interposition dependence.

### Specific Nucleotide Mutations that Contribute to the Co-evolutionary Patterns

We estimate the *CoEvol* values separately for each PWM *M* and for each pair of positions (*i,j*) within the PWM. We have further decomposed this overall measure of co-evolution to estimate the *CoEvol* value for a specific nucleotide-pair (*u,x*) transitioning into another nucleotide-pair (*v,y*) along a specific branch *b* of the evolutionary tree (see Methods). We computed the *CoEvol* values for all combinations of PWM, position-pairs, branch, and quadruples (*u,v,x,y*), thus resulting in ∼8 million *CoEvol* values for the *Foreground*. We also performed this procedure for the 100 *Shuffle* control sets. Each *Foreground CoEvol* is then assigned an empirical p-value based on the corresponding 100 *Shuffle CoEvols*. These p-values are then corrected for multiple testing using the Storey-Tibshirani procedure implemented in R (http://www.r-project.org/). Below, we only consider the *CoEvol* values with a nominal p-value <0.01 (i.e. all 100 *Shuffle CoEvol* values were smaller than the *Foreground CoEvol*). This p-value corresponds to a False Discovery Rate of 16%. Out of 8 million *CoEvol* values, 321162 cases qualify given this threshold. We found that among the 321162 significant cases, scopes 1 through 8 occur at a monotonically decreasing fraction –0.25, 0.17, 0.15, 0.12, 0.1, 0.08, 0.07, and 0.06, consistent with the analysis above. Also consistent with our analysis above, we found that particular PWMs or families do not dominate these significant cases.

Next, we investigated the relative abundance of co-evolving nucleotide-pairs. In each of the significant cases above, we noted the nucleotide quadruple (*u,v,x,y*) where a transition from base *u* to base *v* at one position correlates with a transition from base *x* to base *y* at another position of the PWM along some tree branch. This result can also be viewed as a nucleotide-pair (*u,x*) transitioning to a nucleotide-pair (*v,y*). We only consider the 301697 cases where either u ≠ v OR x ≠ y corresponding to 240 possible quadruples. We found that 28 quadruples are significantly enriched (see Methods). The most represented quadruple is CTGG, which corresponds to a CG to TG transition. This transition is the well-established conditional hyper-mutation of methylated Cytosine (when followed by a Guanine) to a Thymine [26]. Likewise, the quadruple CCGA, corresponding to the CG to TG mutation on the reverse strand, is also among the 28 enriched quadruples. However, the CG hyper-mutability applies only when C and G are adjacent. Indeed the cases involving CTGG or CCGA occur overwhelmingly in scope 1. Neither of the two quadruples is enriched when scope 1 is excluded from the analysis.

There are at least two possible mechanisms underlying the co-evolving quadruples as detected by our approach. The first is conditional hyper-mutability, such as CG to TG, where a specific nucleotide *u*, when followed by *x* is hyper-mutated to *v*. The second mechanism is that of preferred nucleotide-pairs, where (*u,x*) and (*v,y*) are preferred to (*u,y*) or (*v,x*). In the case of hyper-mutability, the mutation in one direction is more likely than in the other direction. For instance, relative to CG to TG transitions, a TG to CG transition should be rare. Indeed the two quadruples TCGG (TG to CG) and CCAG (CA to CG) are not enriched, with ranks 142 and 147 out of 240 quadruples. To study preferred nucleotide-pairs, we tested the extent to which the nucleotide-pair transitions are symmetric, i.e. both (*u,x*) to (*v,y*), as well as (*v,y*) to (*u,x*) transitions are highly represented. We call the two quadruples (*u,v,x,y*) and (*v,u,y,x*) *reciprocal*.

We ranked each of the 240 quadruples by their representation in the significant cases. For each of the 120 *reciprocal* pairs of quadruples, let *r* be the higher (near the top) of the 2 ranks. The difference in the two ranks is expected to be uniformly distributed between 1 and 240-*r*. Based on this assumption, we computed the z-score of the difference in ranks as *(actual difference – expected difference)/standard deviation*. We found that higher the rank *r*, the smaller the difference is in the two ranks. The Kendall’s Tau correlation between the two quantities for the 120 *reciprocal* pairs was 0.26 (p-value 2.3e-05). Thus, there is a reciprocal relationship among highly represented quadruples, consistent with the ‘preferred nucleotide-pairs’ mechanism. However, we note that these are very general observations and a more detailed analysis is required to characterize the preferred nucleotide-pairs for specific PWMs at specific positions.

## Discussion

Here we have reported a novel methodology for assessing co-evolving positions within TF binding sites using the inferred patterns of evolutionary changes at the positions. Relative to a stringent control we found that there is a prevalence of co-evolving position-pairs within the mammalian binding sites. We found that with increasing distance between positions the tendency to co-evolve decreases. This observation is consistent with few known TF-DNA structures [20], [21], which reveal localized interactions between a TF residue and DNA bases. We found that structurally related TFs exhibit relatively similar patterns of interposition dependence. In particular, consistent with the structural organization of zinc finger TFs, we found that the interposition dependence in this family occurs predominantly within scopes 1 through 3. We also found that co-evolving positions tend to be evolutionarily more conserved, suggesting a greater functional constraint.

Earlier work on a similar problem considered the detection of co-evolving positions within and between protein domains [27]. We previously reported an approach to the specific problem addressed here, wherein we inferred the ancestral states based on maximum parsimony and used those inferred ancestral bases to estimate co-evolution [19]. The approach presented here is a methodological improvement over our previous approach in several important aspects. First, our evolutionary inferences here are based on a more robust, *ML* approach [28]. Second, unlike earlier studies, we use a highly stringent *Shuffle* control, which appropriately controls for the compositional and evolutionary properties of individual *cis* element positions and is likely to yield fewer false positives. Several analyses presented here, most notably the analysis of specific prevalent quadruples, are novel.

There are a few potential sources of errors in our analysis. First, our analysis by necessity is based on putative binding sites. However, our reliance on stringent PWM matches within 1 kb human promoters aligned without gaps in 5 mammalian species is likely to minimize the false positives. Second, multiple genome alignment is likely to be error prone and for a small fraction of binding sites, turnover events [29] will render the multiple alignments meaningless. Also in this regard, a comparative genome-scale TF binding study by Schmidt et al. based on liver ChIP-seq for two TFs in three mammalian and two non-mammalian species suggested that in addition to lineage-specific binding site turnover, a majority of binding sites for two TFs in liver are species-specific [30]. Although species-specific sites cannot be used to investigate co-evolution of positions within binding sites, our model can accommodate turned-over sites if the turnover event can be ascertained. Consideration of such events could also improve the sensitivity of our model; conversely not accounting for them may lead to loss of *CoEvol* significance rather than increasing the risk of getting a false signal. Finally, we have estimated the phylogenetic parameters based on concatenated set of multiple alignments for all ∼20,000 gene promoters. Local variations in these parameters will introduce errors in our inference. However these errors will in general obscure the co-evolution signal and are not likely to yield false positive detection of co-evolution.

We were able to interpret some of our specific findings based on limited literature survey for a few TFs. For instance, zinc finger TF *Staf* comprises seven fingers, each recognizing three to four nucleotides. The cascade-like pattern of short-range (within-finger) dependence in *Staf* (Figure S1) is reminiscent of multiple fingers. Consistent with DNA recognition properties of zinc finger proteins, we found that in this family as a whole, there is a significant enrichment of scopes 1 through 3, relative to other families (Fisher’s exact test p-value = 0.02). In the case of nuclear receptor *Ar*, the dependence pattern (Figure S1) mostly assorts in the two known half sites [31]. The ETS family, with known TF-DNA structure for member SAP1/Elk4 [20], is characterized by GGA core in its DNA recognition motif. We detected all three pairs of positions within the core as co-evolving – these are indicated in rows 3, 4, and 6, of the eight interdependent position-pairs for Elk4, in Figure S1. The DNA binding consensus for tumor suppressor *p53* in JASPAR is ccggACATGC**C**C GGGCAT**G**T, with 2 inverted repeats – positions 5–12 and 13–20. Our analysis revealed 2 dependencies at scope = 8. One is between positions 6 and 14 (underlined) and another one between positions 11 and 19 (bold large font). It is interesting to note that the two long-distance dependencies are symmetric with respect to the inverted repeats.

Our observation that the *CoEvol* values for the *RandomContext* are significantly greater than those for *Random* control indicates an underlying mutational dependency in the mammalian genome, at least in the promoter region. This result has been observed previously by Siepel and Haussler [24]. They remarked that while the CpG hyper-mutation effect is pronounced in the mammalian non-coding regions, there is a more complex pattern of context-dependent substitution, comprising a variety of subtle effects [24]. This remark is consistent with our findings based specifically on TF binding sites. While context-dependent substitutions (CpG and others) are likely to contribute to our overall observations, our analysis suggests that an additional alternative mechanism, namely a preference for specific nucleotide combinations in *cis* elements, is likely to play a role in correlated mutations. Even though the interposition dependence is more prevalent for adjacent nucleotides, which is compatible with context-dependent substitution mechanism, the majority of detected interposition dependencies are between non-adjacent positions. A closer investigation of the predictions may provide specific insights into TF-DNA interactions, and may also guide the efforts to model the DNA binding specificity by incorporating the interposition dependencies [7], [8].

## Materials and Methods

### Conserved *cis* Elements in Human Promoters

We obtained 1 kb promoter sequences for the 20835 human RefSeq genes [23] from UCSC database (hg18, genome.ucsc.edu). We searched the promoter sequences for matches to each of the 79 vertebrate PWMs in JASPAR (version 3) [22]. The PWM matches were obtained using a previously published PWMSCAN tool [32] with a p-value threshold of 1.0e-09 corresponding to a random expectation of one hit every ∼8 kb. Genome-wide set of alignments for Human, Chimpanzee, Mouse, Rat, and Dog was downloaded from UCSC. Among the initial matches for the 79 PWMs we retained up to 1000 highest scoring matches (lowest p-values) and then considered only those that were contained within a gapless region of the multiple alignments. Fifteen PWMs had no match qualifying these two criteria, thus our analysis is based on 64 PWMs. The average number of matches per TF was 237.

### Estimating Co-evolution

#### Estimating the likelihood of a multiple alignment column

Consider PWM *M* with *N* genome-wide matches and position *i* within the PWM. There are *N* instances of position *i*, each associated with a multiple alignment column. Let *T* be the evolutionary tree corresponding to the multiple alignment for an instance of position *i.* Thus, *T* has 5 leaf nodes and 4 internal (or ancestral) nodes including the root (Figure 1). Let *A* be the set of 4 ancestral nodes, where the bases are unknown. At each of these 4 nodes, any of the 4 bases could occur. We allow for complete uncertainty in *A*, optimizing over all 4^4^ (256) possibilities. For clarity in the equations below, to describe quantities involving contributions over all 256 possibilities, we use the subscript *p* in a generic context where *A_p_* denotes the *p^th^* possibility. We use the superscript *s* in a specific context to talk about *A^s^* as a specific ancestral node assignment among the 256 possibilities. Note, these are only notational distinctions, and for *p* = *s*, *A_p_* = *A^s^*. Given (1) the tree topology for the five species, (2) an ancestral node assignment *A* for each of the four ancestral species, and (3) the probability of bases at the root and substitution rate matrix for each branch of the tree, the likelihood of *T* can be estimated as the product of the root base probability and all transition probabilities along the branches [28]. In the absence of an *A^s^*, the tree likelihood can be estimated by marginalizing *A,* i.e, by summing over all 256 possible node assignments as
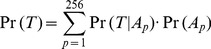
(1)


Given the set of trees for all *N* instances, some of the 256 assignments for A would likely explain the data better than others. We use the data to determine which assignments would be more probable as follows. Let *T^k^* be the tree corresponding to *k^th^* instance of position *i*, *1≤ k ≤ N*. We first estimate the weight of a specific ancestral node assignment *A^s^* as
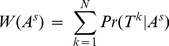
(2)


We do this for all *s*, *1≤ s ≤256*.

Finally, for all *s*, we estimate the probability of the specific ancestral node assignment *A^s^* by normalizing its weight by total weight as
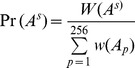
(3)


The denominator in equation (3) represents the sum total of weights for all 256 assignments. Substituting Pr(*A_p_*) in equation (1) by Pr(*A^s^*) in equation (3) (for all *p* and *s*; *p* = *s*) provides an estimate for the likelihood of the tree for a particular instance of position *i*. Thus, a specific strength of our framework is that we consider all possible 256 solutions for *A* in direct relation to their likelihood as estimated by the data.

To illustrate the concepts above, we use the example outlined in Table 1. Consider a PWM with 5 genome-wide matches i.e., TF with 5 putative binding sites. Also consider only two species, so tree *T* has two leaf nodes and only one internal node, the root. Thus there are 4 possible (as opposed to 256) ancestral node assignments: A, C, G, and T. In Table 1, each entry in the 5 columns for “Sites” (numbers in blue) corresponds to Pr(*T^k^|A^s^*) in equation (2). The table shows that each binding site could have any of the node assignments, but with different probabilities. There is no assumption of a fixed assignment for all sites, or even a single site. For each assignment *A^s^*, probabilities over all sites are aggregated to calculate *W*(*A^s^*) and the normalized weight, Pr(*A^s^*), per equations (2) and (3). For example, for *A^s^* = ‘T’ (second row in table), *W*(‘T’) = 0.01+0.0007+0.009+0.08+0.1 = 0.1997 (next to last column, second row). The sum of all entries in the *W*(*A^s^*) column is 0.77098. Thus Pr(‘T’) = 0.1997/0.77098 = 0.259021 (last column, second row). Since Pr(‘C’) is the highest, ‘C’ emerges as the most preferred assignment overall. Note, however, that this does not mean that ‘C’ is the most preferred for all sites – indeed for site 2, ‘A’ is more preferable, and for site 5, ‘T’. Even though all other sites prefer ‘C’, note also that ‘C’ is not a fixed assignment for any site – other assignments are possible, albeit less likely.

**Table 1:** Example to illustrate the concept of normalized weight (or probability) of ancestral assignments.

Ancestral node assignment(*A^s^*)		Sites (*k*)					*W*(*A^s^*)	*Pr*(*A^s^*)
		1	2	3	4	5		
1	A	0.001	0.05	0.0003	0.003	0.01	0.0643	0.0834
2	T	0.01	0.0007	0.009	0.08	0.1	0.1997	0.259021
3	G	0.00008	0.003	0.006	0.004	0.0009	0.01398	0.018133
4	C	0.023	0.018	0.065	0.3	0.087	0.493	0.639446

We present a simplified situation of two species related through a common ancestor, where the evolutionary tree has just one internal node representing the ancestor, with four possible ancestral assignments. For a sample PWM with 5 sites aligned over the two species, we provide representative values (in blue) for the probability of the tree corresponding to each site given a particular ancestral assignment. From these we work out the overall probability of an ancestral assignment given the data (last column). For details, see the text in the Materials and Methods section that references this table.

#### Estimating the likelihood of a multiple alignment column-pair

The above treatment for a single position can be extended naturally to a pair of positions, *i* and *j*, associated with trees *T_i_* and *T_j_* respectively, to estimate the joint likelihood of a pair of positions within a PWM match (in other words, an (*i,j*) instance). Equation (1) can be extended as
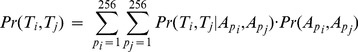
(4)


Note that 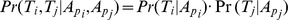.

However, 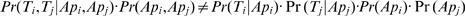
because 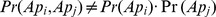. This inequality can be illustrated as follows. As in our example in Table 1, consider position *i* with an ancestral assignment of ‘C’ that occurs at instances of *i* (i.e. the *i^th^* positions over all sites) with certain probabilities. Similarly, consider position *j* with a different set of probabilities for ‘C’ over all instances. The joint assignment ‘CC’ would be preferable only if ‘C’ at position *i* frequently co-occurs with ‘C’ at position *j*. Thus, 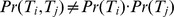
_._

Equation (2) is extended likewise:
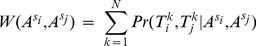
(5)



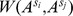
 represents the joint weight for a pair of specific ancestral assignments pertaining to position pair (*i,j*). Both *s_i_* and *s_j_* can be any one of 256 assignments so a total of 256*256 joint weights are evaluated.

By a similar extension of equation (3), we have
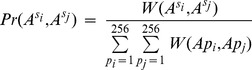
(6)


In general, assignment pairs that tend to evolutionarily track together have a joint weight that is relatively higher than the product of their independent weights. Such assignment pairs suggest coordinated patterns of evolution between positions *i* and *j*.

#### Estimating the co-evolution of a pair of positions

An enrichment of Pr(*T_i_,T_j_*), relative to the product of the two independent likelihoods – 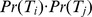, is inferred to indicate the co-evolution of positions *i* and *j*. However, the co-evolution of a pair of positions cannot be inferred based only on a single binding site instance. We aggregate over all *N* instances the log-likelihood ratio of the joint likelihood and the product of independent likelihoods as a measure of co-evolution between positions *i* and *j* of PWM *M*:
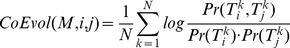
(7)


#### Comparison of CoEvol values for Foreground and RandomContext

We noticed that the *CoEvol* distributions of *Foreground* and *RandomContext* were not significantly different from each other. Upon close inspection, we found that this was because *Foreground* and *RandomContext* differed significantly in their distributions of tree likelihoods, likely due to differences in the conservation properties of binding sites and random positions in the promoters. Distributions for both the numerator and denominator in equation 5 were left-shifted in *RandomContext* as compared to *Foreground*. We hypothesized that the overall lower conservation for the *RandomContext* sites would lead to lower value of denominator, which in turn may bias the *CoEvol* values. To control for this, for each *(M,i,j)* triplet in the *Foreground*, for each of the *N* instances, we randomly selected position-pairs from *RandomContext* sites with scope = *j - i* such that the product of tree likelihoods at the positions in the selected pair was “similar” to 
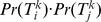
 for the *Foreground* observation. More specifically, values for 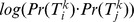 ranged between −16 and 0. We divided this range into 16 equal-sized bins. For each position-pair in the *Foreground*, we then randomly selected a pair from *RandomContext* with 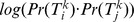 value in the same bin as that for the *Foreground* pair. We used the *N* selected pairs according to equation 5 to compute *CoEvol(M,i,j)* for the *RandomContext*. Note that all *RandomContext CoEvol* values used in our analysis as well as depicted in Figure 2 are generated after correction for tree likelihood distributions. As with *Shuffle*, this procedure was repeated 100 times to obtain 100 *RandomContext CoEvol* values for each *Foreground CoEvol* value. Although tree likelihood distributions were left-shifted in *RandomContext*, generating a sufficiently large *RandomContext* dataset yielded enough values to help contrast *Foreground* observations, even ones with high
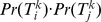
.

#### Estimating the parameters of the phylogenetic model

The phylogenetic model consists of the substitution rate matrix *Q*, equilibrium base frequencies *π*, binary tree *τ*, and branch lengths *β* [24]. HKY model parameterization was used for *Q*. Based on the 5-species multiple alignment of 1 kb human promoters, we used PAML software [33] to compute the maximum likelihood estimate of the phylogenetic model. Finally using the optimized parameters for *Q*, we calculated a transition probability matrix for each branch of *τ*, following the approach described in [34] (omitted here for brevity).

### Estimating the Likelihood of Specific Quadruple at a Specific Combination of Motif, Positions, and Branch

As before, consider PWM *M* with *N* matches, and position *i* within the PWM. Additionally consider a particular branch *b, 1≤ b ≤8*, of the rooted binary tree for 5 mammalian species. We use the notation *t^k^* to denote the nucleotide transition along branch *b* at position *i*. For any instance *k*, *1≤ k ≤ N* and for the corresponding tree *T^k^*, if nucleotide *u* transitions to *v* along branch *b*, we have *t^k^* = *uv*. Not all trees would carry a specific transition. We define the indicator function *I_uv_*(*t^k^*) such that
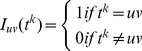
(8)


Thus for a tree *T^k^* having any one of 256 possible ancestral assignments, *I_uv_*(*t^k^*) = 1 if and only if along branch *b* of the tree nucleotide *u* transitions to *v*. Then the likelihood of this particular transition from *u* to *v*, can be estimated as the ratio of conditional likelihood of the tree (conditioned on the specific transition) and the unconditional tree likelihood. Over all *N* instances the average propensity of observing an *u* to *v* transition along branch *b*, denoted by Pr(*u,v*), is estimated as
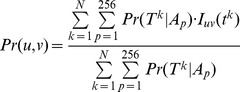
(9)


Let 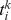 and 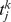 denote the nucleotide transitions along branch *b* at positions *i* and *j* respectively. Equation (9) can now be extended to estimate Pr(*u*,*v*,*x*,*y*), or the joint transition of nucleotide pair (*u*,*x*) to (*v*,*y*) at position pair (*i*,*j*) of PWM *M* along branch *b*:
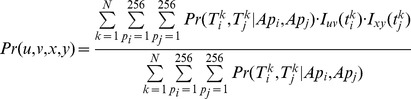
(10)


Comparing the numerators in equation (9) and (10) we note that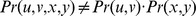.

The *CoEvol* value for a specific nucleotide-pair (*u,x*) transitioning into another nucleotide-pair (*v,y*) at position pair (*i*,*j*) of PWM along a specific branch *b* is then given by
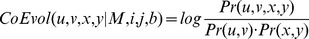
(11)


### Enriched Representation of Specific Quadruples

If there are a total of *N* significant cases and *M* quadruples, the expected mean and standard deviation of the number of times an arbitrary quadruple is represented are 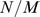 and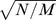. If a quadruple is represented in greater than 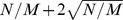, we consider it significantly enriched (nominal p-value <0.05).

## Supporting Information

Figure S1 **Co-evolving position-pairs.** All 315 position pairs that were deemed to be co-evolving with FDR ≤0.05. The positions are listed by PWMs and are 0-based. Each row shows an interdependent position-pair (end-points, represented by light blue squares) connected by intervening positions (link, shown by dark blue squares). Grey squares represent positions outside of interdependent position-pair and white squares are beyond the length of the PWM.
(PDF)

Table S1 **The list of JASPAR vertebrate PWMs used in our study.** ID is the same as in JASPAR database. # Hits represents the number of qualifying binding sites we recovered per PWM. For 15 PWMs we did not get any binding site data, so these were excluded from further analysis. The PWM length for the 64 PWMs with binding sites is indicated.
(XLSX)

Table S2 **List of 315 co-evolving position-pairs that were deemed significant.** “Scope” and “Begin Position” together define the coordinates of the position-pairs on the PWM. For example, Scope = i and Begin Position = j implies that the position-pair is (j, i+j).
(XLSX)

Text Archive S1 **A compressed/ZIP file archive of all binding site data for the 64 PWMs used in our analysis.** The individual files are named per PWM ID, so for instance, “MA0002.hits” corresponds to JASPAR PWM MA0002. This has 333 binding sites, each of length 9. Each binding site is grouped as a set of 5 aligned sequences corresponding to human, chimp, mouse, rat and dog (from first to last). A blank line separates binding sites from each other.
(GZ) Click here for additional data file.
