# Supplementary figures and images for: Correlated Evolution of Positions within Mammalian cis Elements
Source: PLoS One. 2013 Feb 8;8(2):e55521. doi: 10.1371/journal.pone.0055521 (PMC3568137; doi:10.1371/journal.pone.0055521)

[illegible]

[illegible]

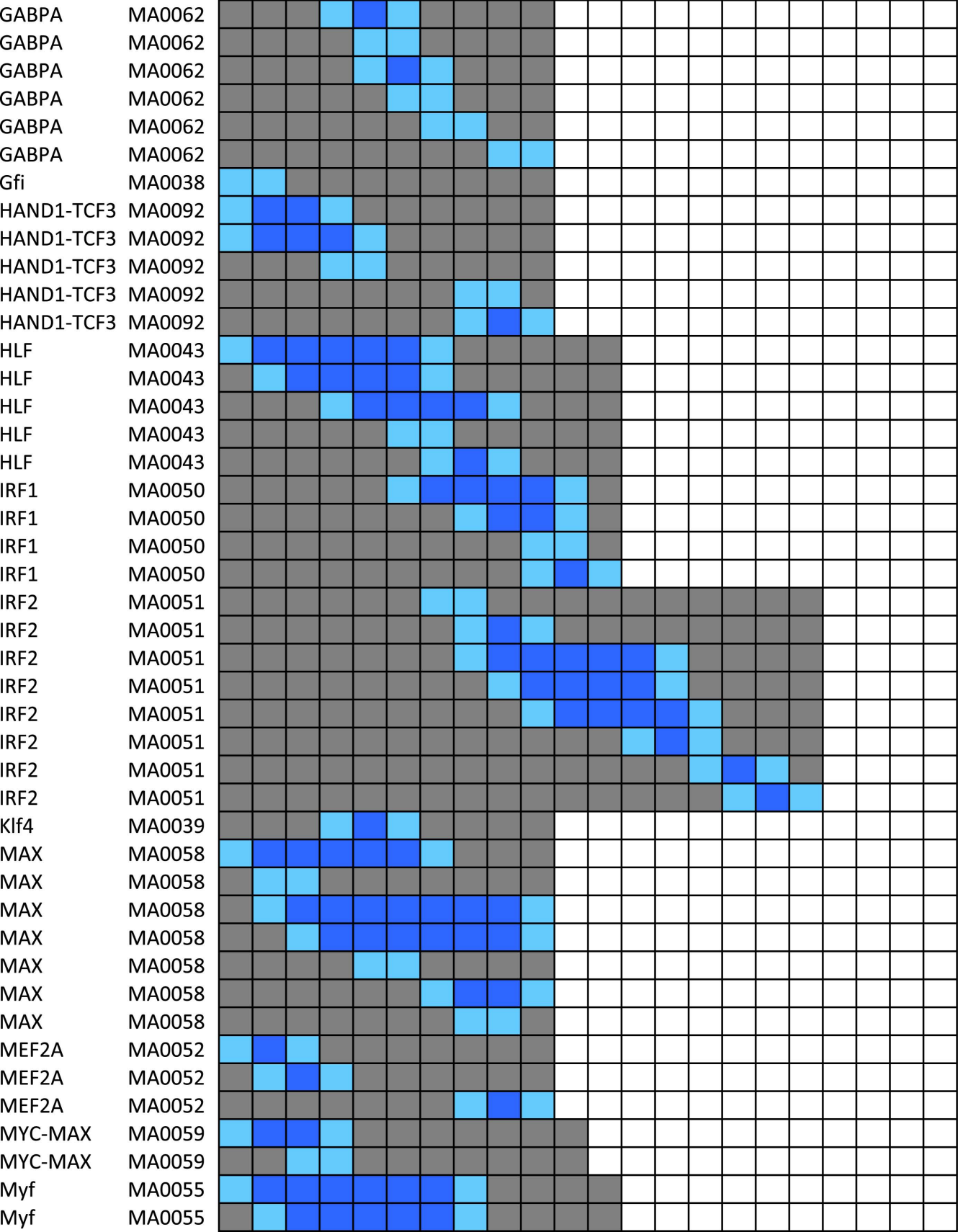

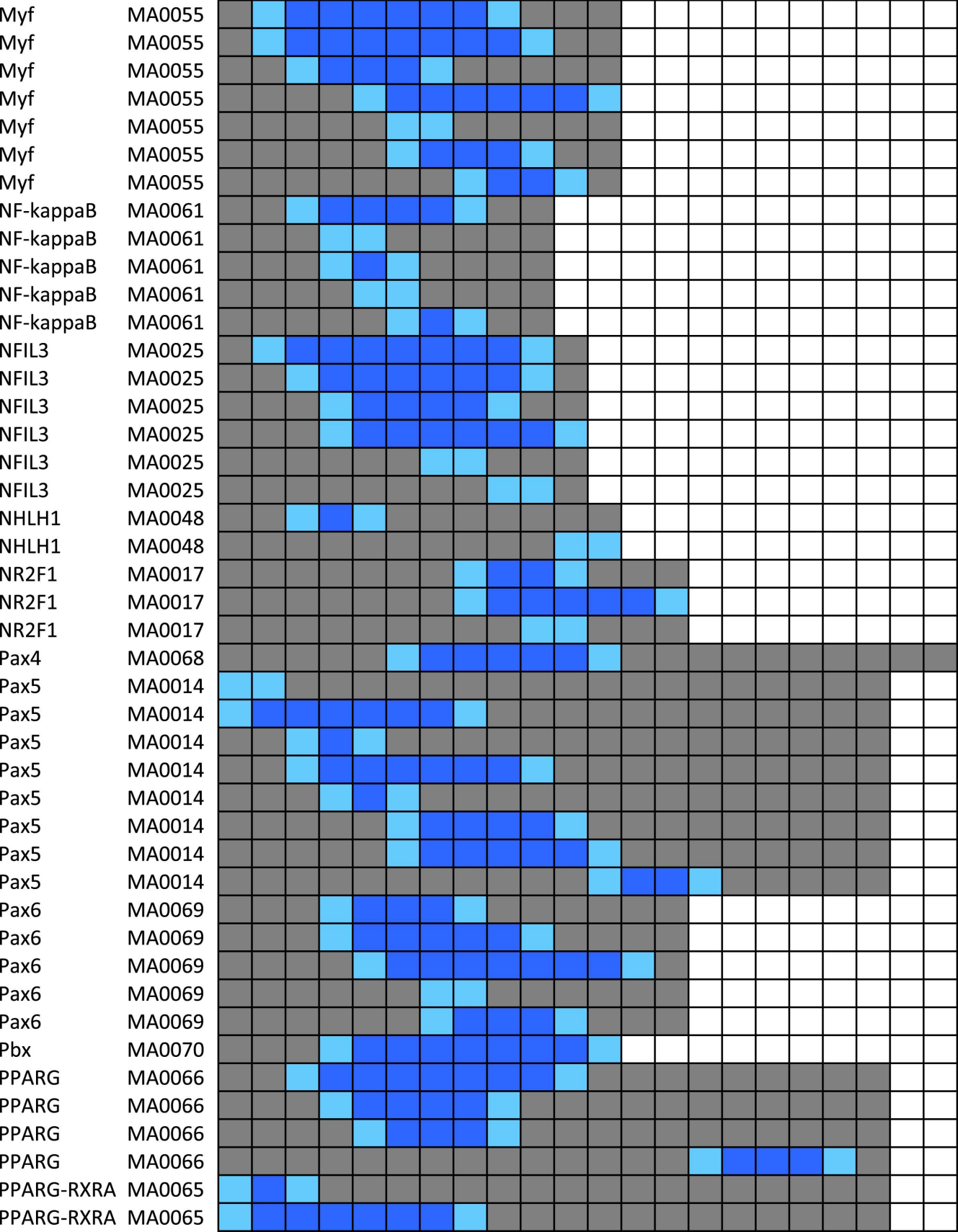

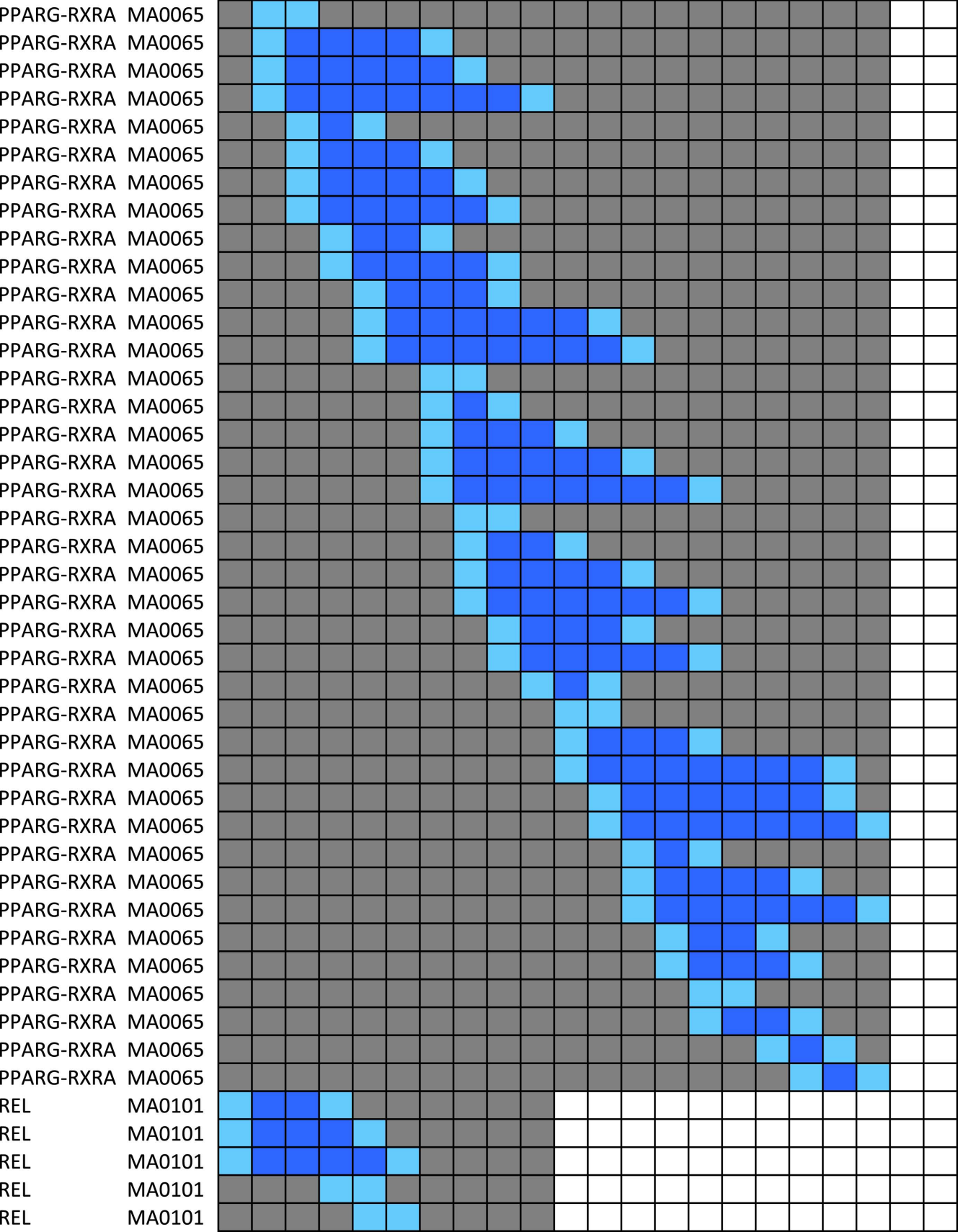

[illegible]

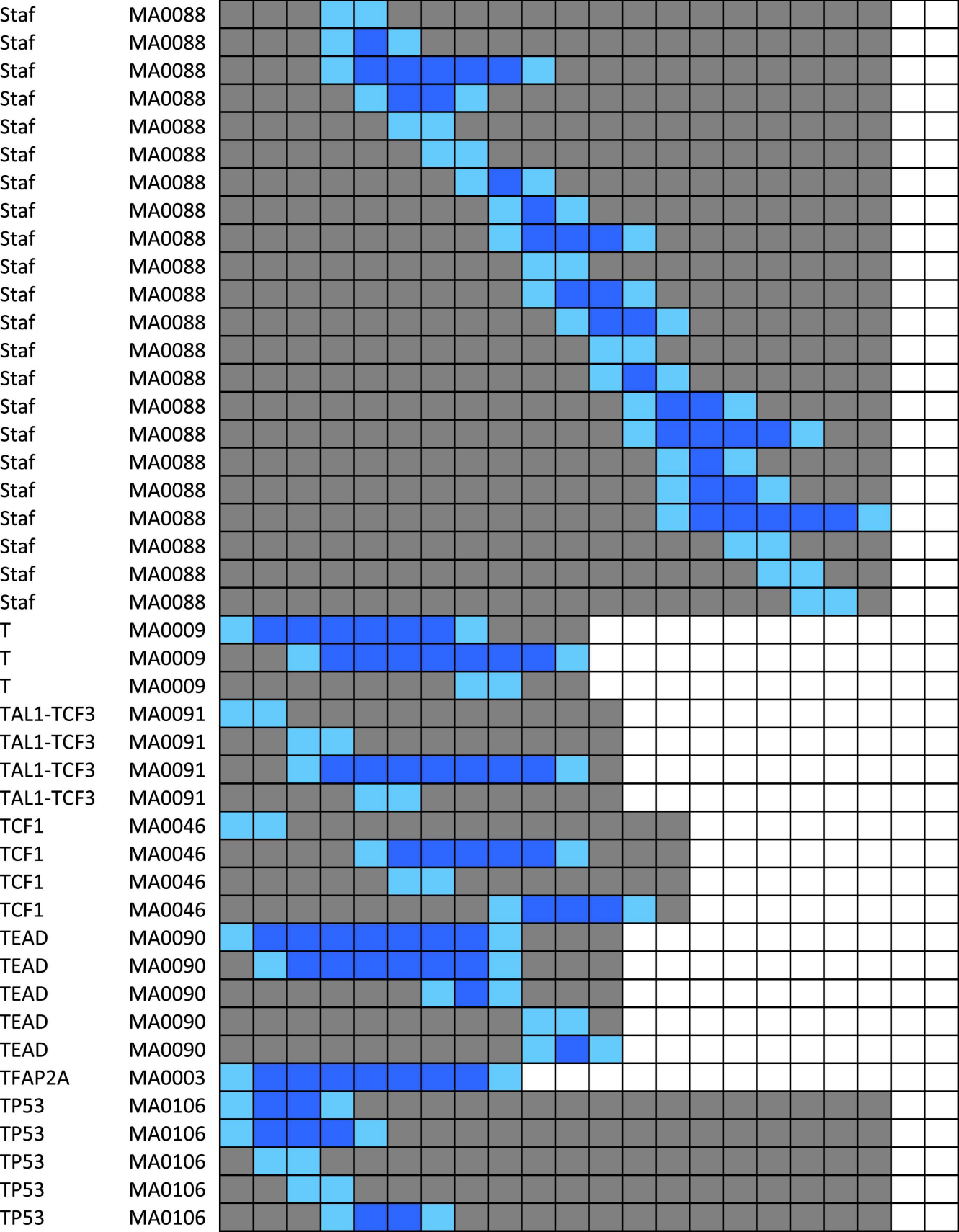

|            |        |  |  |  |  |  |  |  |  |  |  |  |  |  |  |  |  |  |  |  |
|------------|--------|--|--|--|--|--|--|--|--|--|--|--|--|--|--|--|--|--|--|--|
| TP53       | MA0106 |  |  |  |  |  |  |  |  |  |  |  |  |  |  |  |  |  |  |  |
| TP53       | MA0106 |  |  |  |  |  |  |  |  |  |  |  |  |  |  |  |  |  |  |  |
| TP53       | MA0106 |  |  |  |  |  |  |  |  |  |  |  |  |  |  |  |  |  |  |  |
| TP53       | MA0106 |  |  |  |  |  |  |  |  |  |  |  |  |  |  |  |  |  |  |  |
| USF1       | MA0093 |  |  |  |  |  |  |  |  |  |  |  |  |  |  |  |  |  |  |  |
| USF1       | MA0093 |  |  |  |  |  |  |  |  |  |  |  |  |  |  |  |  |  |  |  |
| USF1       | MA0093 |  |  |  |  |  |  |  |  |  |  |  |  |  |  |  |  |  |  |  |
| ZNF42_5-13 | MA0057 |  |  |  |  |  |  |  |  |  |  |  |  |  |  |  |  |  |  |  |

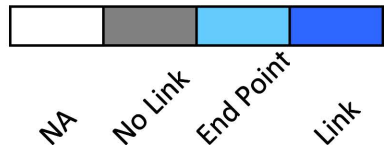

Supplement: Figure S1 — Co-evolving position-pairs. All 315 position pairs that were deemed to be co-evolving with FDR ≤0.05. The positions are listed by PWMs and are 0-based. Each row shows an interdependent position-pair (end-points, represented by light blue squares) connected by intervening positions (link, shown by dark blue squares). Grey squares represent positions outside of interdependent position-pair and white squares are beyond the length of the PWM. (PDF) [file pone.0055521.s001.pdf]
